# Sodium Benzoate, Potassium Sorbate, and Citric Acid Food Preservatives Trigger Apoptosis in the Male Reproductive System of Rats

**DOI:** 10.1155/bmri/8837003

**Published:** 2025-09-10

**Authors:** Marziyeh Haghshenas, Seyyed Sajad Daneshi, Ahmadi Hassan Nategh, Samaneh Bina, Fateme Esmaeilpoor, Razieh Bagheri, Mohammad Javad Khoshnoud, Azad Salimi, Seyedeh leili Asadi-Yousefabad, Marzieh Rashedinia

**Affiliations:** ^1^ Department of Pharmacology and Toxicology, School of Pharmacy, Shiraz University of Medical Sciences, Shiraz, Iran, sums.ac.ir; ^2^ Department of Anatomy, School of Medicine, Shiraz University of Medical Sciences, Shiraz, Iran, sums.ac.ir; ^3^ College of Veterinary Medicine, Shanxi Agricultural University, Taigu, Shanxi, China, sxau.edu.cn; ^4^ College of Animal Science and Veterinary Medicine, Shiraz University, Shiraz, Iran, shirazu.ac.ir; ^5^ Department of Biology, Marvdasht Branch, Islamic Azad University, Marvdasht, Iran, azad.ac.ir; ^6^ Student Research Committee, Shiraz University of Medical Sciences, Shiraz, Iran, sums.ac.ir; ^7^ Department of Molecular Medicine, School of Medicine, Shiraz University of Medical Sciences, Shiraz, Iran, sums.ac.ir

**Keywords:** apoptosis, citrate, food preservatives, male reproductive system, potassium sorbate, sodium benzoate

## Abstract

The safety of food additives has received significant attention. While individual additives used within specified limits are considered safe, examining the effects of food additives is essential, especially when they are ingested in combination or when consumption exceeds acceptable daily intake levels. In this study, the effects of three common food preservatives—sodium benzoate (SB), potassium sorbate (PS), and citric (Cit) acid—on biochemical markers and histopathology of the male reproductive system were evaluated. Thirty‐six male Sprague–Dawley rats received the no observed adverse effect level (NOAEL) doses of SB, PS, and Cit alone or in combination in their drinking water for 8 weeks. Blood samples and the epididymis and testes were surgically collected for analysis. Results showed significant decreases in testicular weight, reduced sperm count and viability, and increased immotile sperm in the SB, PS, and SB + PS groups. These changes were associated with notable decreases in plasma testosterone levels in all treated groups. Elevated lipid peroxidation levels, increased Bax/Bcl‐2 ratios, and caspase‐3 activity in the combined groups demonstrated induction of apoptosis in testes. Additionally, histopathological analysis revealed degenerative changes in testicular structure and disruption of spermatogenesis. In conclusion, the combined use of these preservatives may lead to reproductive issues in rats, raising concerns about their potential effects on human health.

## 1. Introduction

The transformation in dietary habits has prompted the food industry to incorporate various additives that enhance shelf life and improve physicochemical properties of processed foods [1]. Although many food additives are categorized as “generally recognized as safe” (GRAS), comprehensive understanding of their health implications remains limited [2]. Sodium benzoate (SB) (E211), potassium sorbate (PS) (E202), and citric (Cit) acid (E330) are commonly utilized preservatives to inhibit microbial growth in food products. SB is approved by the FDA as a food preservative with an acceptable intake of 0–5 mg/kg body weight [3]. Studies have demonstrated its negative impacts on liver and renal function, contribution to gastrointestinal issues, and associations with reproductive hormone alterations [4–7].

PS has an acceptable daily intake of 25 mg/kg body weight established by JECFA [3].

Research indicates that PS can induce genotoxic effects in human lymphocytes and cause DNA strand breakage with potential carcinogenic effects [8]. Additionally, PS exposure activates inflammatory pathways and affects cell cycle processes in liver tissue [9, 10].

Cit acid, while naturally occurring and FDA‐approved, has been shown to cause toxicity in dental cells, hepatocyte alterations, and chromosomal damage [11]. Studies also suggest its role in obesity and glucose intolerance [12].

Previous research has demonstrated that food additives significantly impact reproductive systems, with recent studies showing that PS alters semen parameters and testicular architecture in male rats [13], while SB exacerbates testicular toxicity through oxidative stress mechanisms [14].

While individual effects of these preservatives have been studied, their combined impact remains poorly understood. People typically consume multiple preservatives simultaneously through processed foods, potentially leading to synergistic effects that exceed individual acceptable daily intake values [15].

However, ADI values are generally assigned for single substances, and combined exposure can lead to additive toxic effects, which have not been fully characterized in many cases. Therefore, investigating specific preservative combinations helps uncover any unexpected toxicological effects, enabling the development of safer guidelines and regulatory limits for their combined use. This research is aimed at analyzing the effects of Cit acid, SB, and PS, both separately and in combination, on the reproductive system of male rats.

## 2. Methods

### 2.1. Animals and Experimental Design

Thirty‐six male Sprague–Dawley rats were sourced from the Center of Comparative and Experimental Medicine of Shiraz University of Medical Sciences. The study followed the ethical guidelines of Shiraz University of Medical Sciences for laboratory animal care and handling (Ethics Code: IR.SUMS.AEC.1402.051). Following a 1‐week acclimatization period, the rats were allocated into six experimental groups (*n* = 6). There were no exclusions. The rats received oral treatment with the no observed adverse effect level (NOAEL) dose of each food preservative in their drinking water for 8 weeks. The groups included a control group (receiving drinking water without additives), a Cit group treated with 1200 mg/kg of Cit acid in drinking water, a SB group treated with 500 mg/kg of SB in drinking water, a PS group treated with 300 mg/kg of PS in drinking water, a Cit + SB group treated with 1200 mg/kg of Cit acid combined with 500 mg/kg of SB in drinking water, and an SB + PS group treated with 500 mg/kg of SB combined with 300 mg/kg of PS in drinking water. SB at concentrations up to 1% in beverage products is tasteless [7]. In our study, the treatment dose in drinking water was about 0.2%. Additionally, the amount of water consumed by the treated mice was comparable to that of the control group.

### 2.2. Blood Sample Collection and Testis Preparation

At the end of treatment, the animals were administered anesthesia via intraperitoneal injection of ketamine/xylazine at dosages of 100 and 10 mg/kg, respectively. Blood samples were collected through cardiac puncture for serum separation and were subsequently stored at −20°C until analysis of testosterone levels. The epididymis and testes were surgically excised, and their weights were recorded. The left testes were preserved in a 10% formalin solution for future histopathological evaluations. The right testes were promptly transferred to a −80°C freezer for gene expression assays, assessment of total antioxidant capacity, measurement of lipid peroxidation, and evaluation of reactive oxygen species (ROS) production. For sperm analysis, sperm was extracted from the left cauda epididymis.

### 2.3. Body and Reproductive Organ Weights

Body weights were noted before sacrifice; testes and epididymis were removed, cleared of adipose and connective tissues, and weighed. Then, relative organ weights were computed.

Testis and epididymis relative weight was measured by the organ weight index formula [16].

Organ weight index=organ weightbody weight×100.

### 2.4. Semen Characteristics

Sperm motility characteristics and the number of epididymal sperm were assessed under a microscope. The caudal epididymis was minced in warm phosphate‐buffered saline (PBS; 35°C; pH = 7.4) to obtain epididymal sperm. By placing a drop of sperm suspension on a coverslip‐covered glass slide and monitoring the sperm under a 400x magnification Zeiss compound light microscope (fitted with a 35°C hotstage), the motility of the sperm was recorded. Using a light microscope with 200x magnification, 10 *μ*L of diluted epididymal fluid was placed on a Neubauer hemocytometer to determine sperm concentration. After staining with eosin–nigrosin, sperm abnormalities and viability were observed.

### 2.5. Reproductive Hormone

Testosterone was determined using an enzyme‐linked immunosorbent assay (ELISA) kit. The assay was done according to the manufacturer’s instructions.

### 2.6. Markers of Oxidative Stress and Antioxidant Parameters in Testis Tissue

#### 2.6.1. Testicular ROS

The assessment of ROS levels in testicular tissue was performed utilizing the fluorescent probe dichlorodihydrofluorescein diacetate (DCFH‐DA) [17]. A volume of 10 *μ*L of DCFH‐DA was added to 1 mL of homogenized testicular samples, achieving a final concentration of 10 *μ*M, with each sample containing 1 mg of protein per milliliter. The samples were then incubated in the dark at 37°C for 30 min. Following this incubation period, the fluorescence intensity of DCF was measured using a BioTek Synergy HTX Multimode Reader (BioTek, United States), with an excitation wavelength set at 485 nm and an emission wavelength at 525 nm.

#### 2.6.2. Ferric Reducing Antioxidant Power (FRAP) of Testis Tissue

The antioxidant capacity of testicular tissue was assessed by measuring its FRAP [18]. A fresh FRAP reagent was prepared using a 300 mmol/L acetate buffer (pH = 3.6), 10 mmol/L TPTZ (2,4,6‐tripyridyl‐s‐triazine) dissolved in 40 mmol/L HCl and 20 mmol/L ferric chloride in a ratio of 10:1:1. A 100 *μ*L sample of testicular homogenate was combined with 1 mL of the FRAP reagent and incubated at 37°C for 5 min in a dark place. The resulting color intensity was measured at a wavelength of 595 nm with a BioTek Synergy HTX Multimode Reader (BioTek, United States).

#### 2.6.3. Testicular Lipid Peroxidation

The evaluation of lipid peroxidation in testis tissue was conducted utilizing the thiobarbituric acid reactive substances (TBARS) assay [19]. In this procedure, 1 mL of a 10% (*w*/*v*) homogenate of testis tissue in KCl (1.15% *w*/*v*) was combined with 1 mL of thiobarbituric acid solution (0.375% *w*/*v*) and 1 mL of phosphoric acid (1% *w*/*v*, pH = 2). The resulting mixture was subjected to boiling at 100°C for 45 min. Following this, the samples were cooled and centrifuged at 10,000 rpm for 10 min, and the color absorbance was measured at a wavelength of 532 nm.

### 2.7. The Quantitative Real‐Time Polymerase Chain Reaction

Rat testes were examined for the expression of apoptosis‐related genes (Bax, Bcl‐2, and caspase‐3) through real‐time PCR [20]. Total RNA was extracted from 50 mg of tissue using a commercial kit (Parstous, Iran). The quality and quantity of the RNA were assessed with a BioTek Synergy HTX Multimode Reader (BioTek, United States). Complementary DNA (cDNA) was synthesized from the RNA samples using a cDNA synthesis kit (Parstous, Iran). Real‐time PCR was performed utilizing a thermal cycler and the SYBR green detection system (Applied Biosystems, United States). The primer sequences were as follows: Bax: 5 ^′^‐ATGGGCTGGACACTGGACTTC‐3 ^′^; 5 ^′^‐GAGCGA GGCGGTGAGGAC‐3 ^′^; Bcl‐2: 5 ^′^‐TCCTTCCAGCCTGAGAGCAAC‐3 ^′^; 5 ^′^‐GCGACGGTAGCGACGAGAG‐3 ^′^; caspase‐3: 5 ^′^‐GCAGCAGCCTCAAATTGT TGA CAT‐3 ^′^; 5 ^′^‐TGCTCCGGCTCAAACCATC‐3 ^′^; and *β*‐act: 5 ^′^‐ACTATCGGCAATGAGCGGTTCC‐3 ^′^; 5 ^′^‐CTGTGTTGGCATAGAGGTCTTTACG‐3 ^′^. Target gene expression levels were compared to *β*‐actin as a housekeeping gene using the 2^−*Δ* *Δ*CT^ method.

### 2.8. Histopathological Examination

Following the euthanasia of the animals, the testicular tissues were excised and promptly preserved in 10% formaldehyde. Subsequently, the tissues underwent dehydration through a series of increasing ethanol concentrations, followed by embedding in paraffin. For histopathological assessment, Masson’s trichromatic staining (Merck, Germany) was utilized.

### 2.9. Statistical Analysis

GraphPad Prism 6 software (Graph Pad Software Inc., San Diego, CA, United States) was utilized for conducting the statistical analysis. A *p* value of less than 0.05 was deemed statistically significant. The data are expressed as the mean ± SEM. Comparisons of the data were carried out using one‐way analysis of variance.

To analyze the histomorphometric parameters, hierarchical cluster analysis was performed using log2‐transformed signal data. Pearson’s correlation was employed as the distance metric for clustering, utilizing R software (Version 4.3.1).

## 3. Results

### 3.1. Alterations in Organ Weight Index

The relative weight of the testis exhibited a significant decrease across all treatment groups, with the exception of the Cit group, which demonstrated an increase when compared to the control group (*p* < 0.05) (Figure 1a). Conversely, there was a marked increase in the relative weight of the epididymis in all treatment groups when compared to the control group (*p* < 0.05) (Figure 1b).

Figure 1Effects of food preservatives on (a) testicular weight index and (b) epididymis weight index. Data are shown as mean ± SEM, *n* = 6. Cit, citric acid; SB, sodium benzoate; PS, potassium sorbate.  ^∗^ *p* < 0.05 and  ^∗∗^ *p* < 0.01 compared to the control group.

**Figure:**
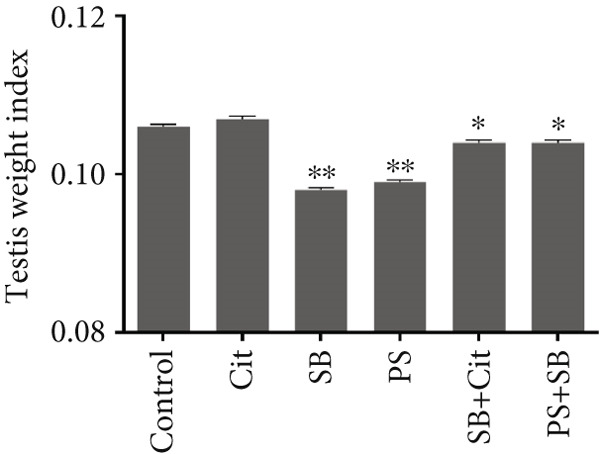
(a)

**Figure:**
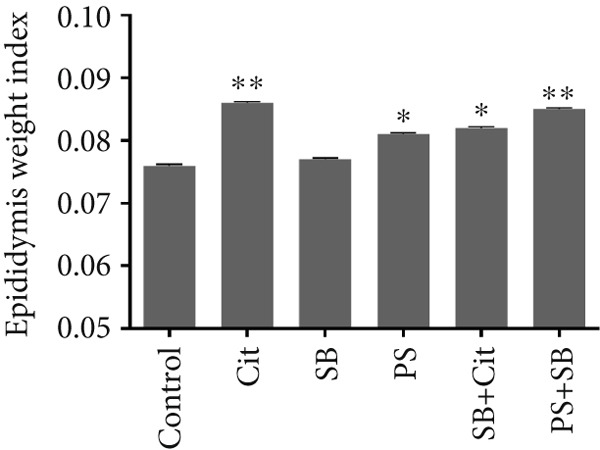
(b)

### 3.2. The Outcomes of Preservative Effects on Sperm Characteristics

In the SB and PS + SB treatment groups, there was a significant decrease in total sperm count compared to the control group (*p* < 0.05) (Figure 2a). Sperm viability was also reduced in the PS and PS + SB groups (Figure 2). Furthermore, the sperm motility in the PS, SB, and PS + SB treatment groups showed a more significant reduction compared to the other groups (*p* < 0.05). In these treatment groups, there was not only a decrease in the count of rapidly moving sperm but also an increase in the number of nonmotile sperm (Figure 2c).

Figure 2Effects of food preservatives on (a) total sperm count, (b) sperm viability, (c) sperm motility, and (d) serum testosterone levels. Data are shown as mean ± SEM, *n* = 6. Cit, citric acid; SB, sodium benzoate; PS, potassium sorbate.  ^∗^ *p* < 0.05,  ^∗∗^ *p* < 0.01, and  ^∗∗∗^ *p* < 0.001 compared to the control group.

**Figure:**
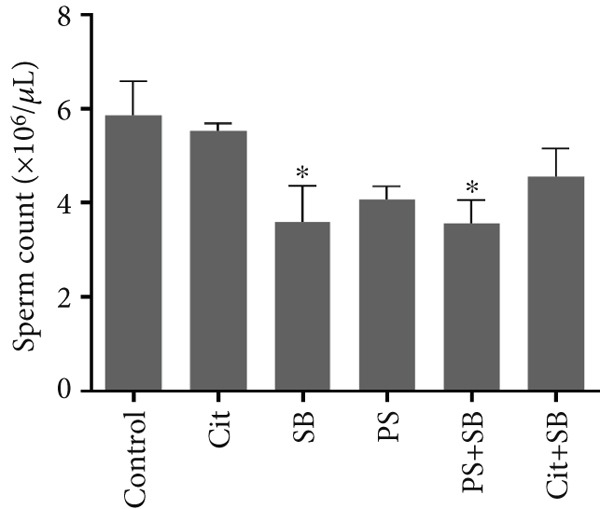
(a)

**Figure:**
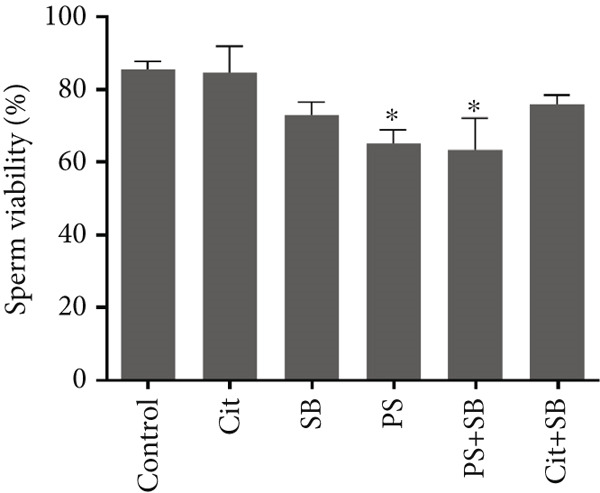
(b)

**Figure:**
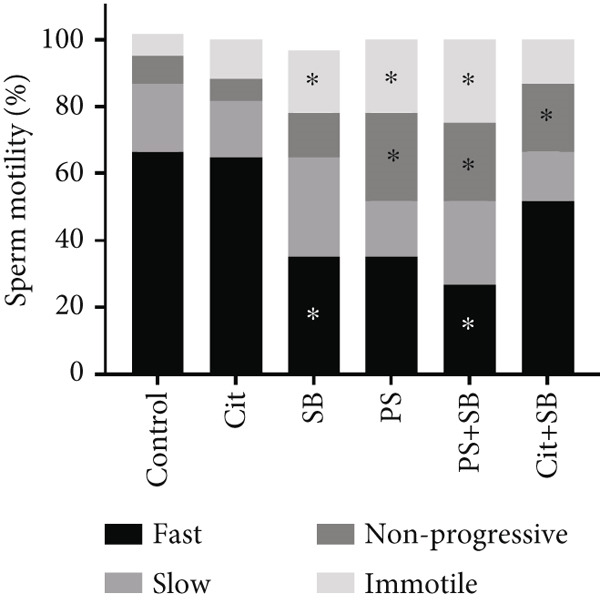
(c)

**Figure:**
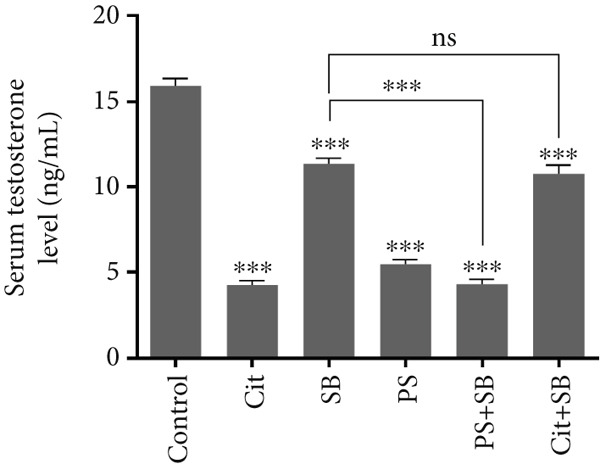
(d)

### 3.3. Fluctuations in Serum Testosterone Levels

The study found a significant decrease in plasma testosterone levels in all treatment groups compared to the control group (*p* < 0.001). The combination group PS + SB showed a greater decrease than the Cit + SB combination group (*p* < 0.001). No significant changes were observed in the SB group compared to the Cit + SB group. Additionally, the PS + SB group had a significant decrease compared to the SB group alone. There were no significant changes between the PS group and the PS + SB group (Figure 2d).

### 3.4. Changes in Markers of Oxidative Stress and Antioxidant Parameter

The biomarkers for oxidative stress in the testis tissue did not show any significant changes in ROS formation in all treatment groups (Figure 3a). The malondialdehyde (MDA) test results indicated a significant increase in lipid peroxidation in PS, PS + SB, and Cit + SB treatment groups (Figure 3b). Additionally, there were no significant changes in antioxidant capacity across any of the treatment groups (Figure 3).

Figure 3Effects of food preservatives on oxidative stress parameters in testicular tissue. (a) Reactive oxygen species (ROS) generation, (b) malondialdehyde (MDA) level, and (c) ferric reducing antioxidant power (FRAP). Data are shown as mean ± SEM, *n* = 6. Cit, citric acid; SB, sodium benzoate; PS, potassium sorbate.  ^∗^ *p* < 0.05 compared to the control group.

**Figure:**
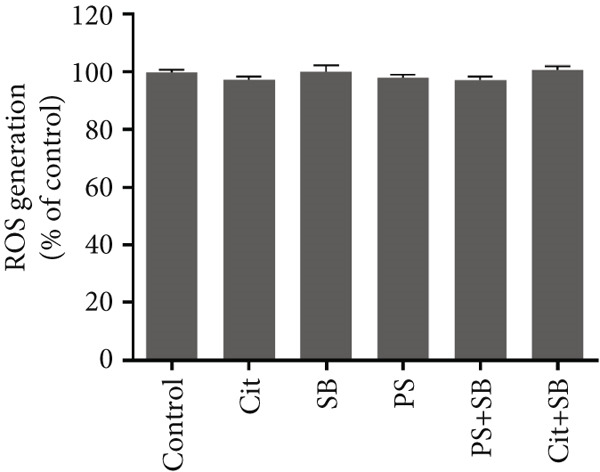
(a)

**Figure:**
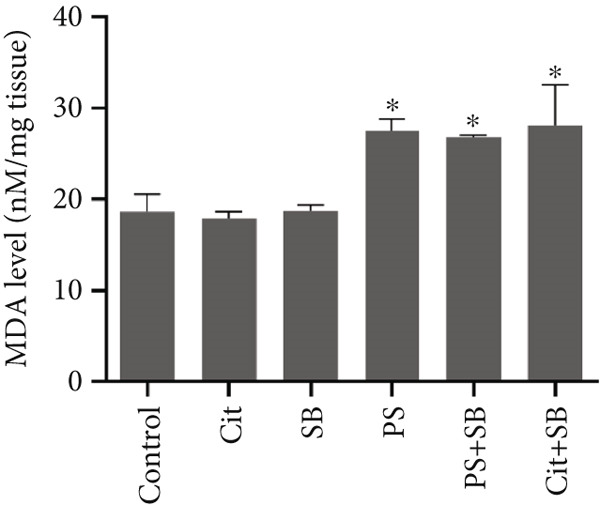
(b)

**Figure:**
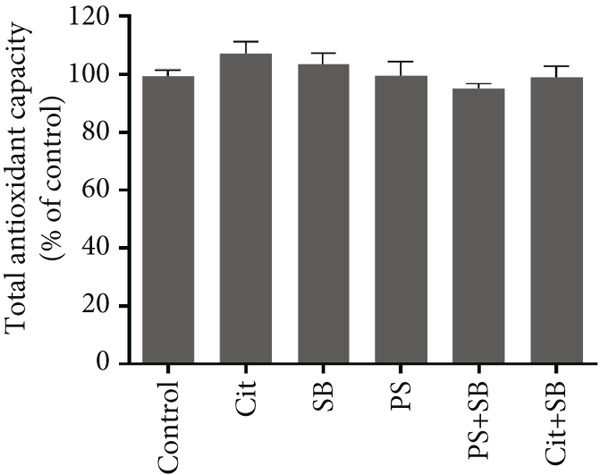
(c)

### 3.5. Effects of Food Preservative Combination on Apoptosis Induction

The expression of genes involved in the apoptosis process was significantly altered in the testis of treated animals. Bax, a proapoptotic gene, was found to be overexpressed in all treatment groups except SB when compared to the control group (*p* < 0.01 and *p* < 0.001) (Figure 4a). On the other hand, Bcl‐2, an antiapoptotic gene, showed a significant increase in the Cit + SB group compared to other groups (*p* < 0.001). It was also increased in Cit, SB, and PS groups (*p* < 0.05 and *p* < 0.01). However, there were no significant changes in Bcl‐2 levels in the PS + SB treatment group (Figure 4b). The Bax/Bcl‐2 ratio was significantly higher in the PS and PS + SB treatments, while the increase in the Cit + SB group was not significant (Table 1). Caspase‐3, another proapoptotic gene, showed a significant increase in the PS and PS + SB groups (*p* < 0.001) (Figure 4c).

Figure 4Effects of food preservatives on mRNA expression levels of apoptotic genes in testicular tissue. (a) Bax, (b) Bcl‐2, and (c) caspase‐3. Data are shown as mean ± SEM, *n* = 6. Cit, citric acid; SB, sodium benzoate; PS, potassium sorbate.  ^∗^ *p* < 0.05,  ^∗∗^ *p* < 0.01, and  ^∗∗∗^ *p* < 0.001 compared to the control group.

**Figure:**
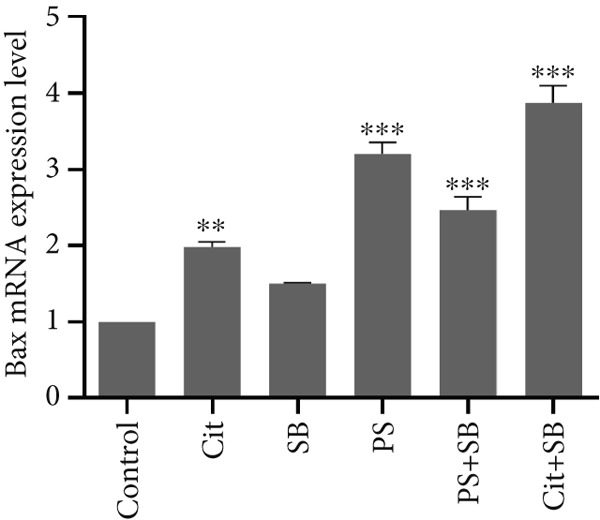
(a)

**Figure:**
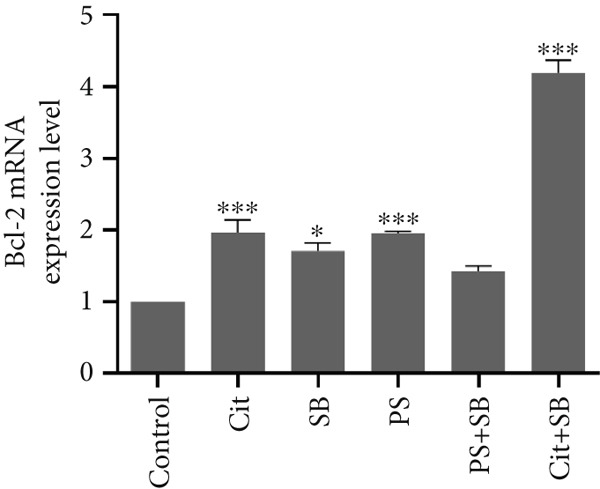
(b)

**Figure:**
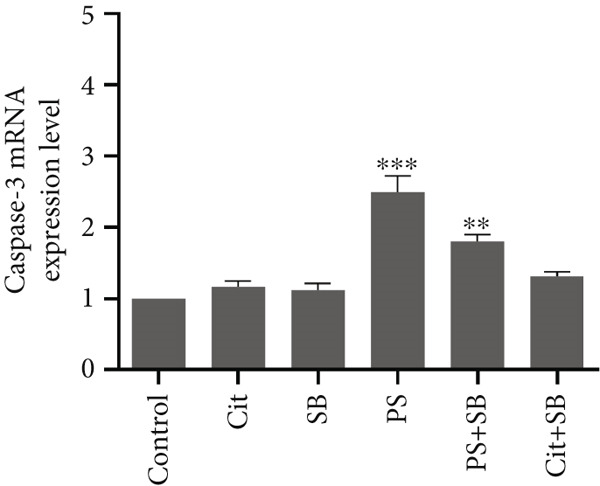
(c)

**Table 1:** Effects of food preservatives on mRNA expression levels of Bax/Bcl‐2 ratio in testicular tissue. Cit, citric acid; SB, sodium benzoate; PS, potassium sorbate.

**Treatments**	**Control**	**Cit**	**SB**	**PS**	**PS + SB**	**Cit + SB**
Bax	1	1.987	1.497	3.200	2.460	3.867
Bcl‐2	1	1.963	1.700	1.957	1.420	4.193
Bax/Bcl‐2	1	1.030	0.890	1.634 ^∗^	1.729 ^∗^	0.930

^∗^ *p* < 0.05 compared to the control group.

### 3.6. Testicular Histopathology and Histomorphometry of Rats Exposed to Preservatives

**Figure 5:**
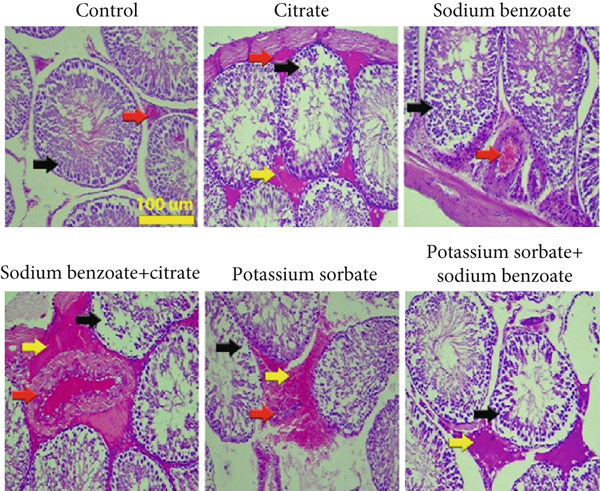
Histological alterations of seminiferous tubules. Blood vessel (red arrow), epithelium of seminiferous tubules (black arrow), hemorrhage (yellow arrow), and Masson’s trichromatic staining.

Based on Masson’s trichromatic staining, the control group showed normal blood vessels (red arrow) and seminiferous tubule epithelium (black arrow) (Figure 5). In addition to the fragmented arrangement of seminiferous tubules epithelium (black arrow), hemorrhage (yellow arrow) was observed in those rats that received Cit. Also, the SB group revealed fragmented epithelial cells (black arrow) along with dilated blood vessels (red arrow). Moreover, similar to the PS group, in the group that received SB and Cit, abnormal cellular attachment and fragmented epithelial cells of seminiferous tubules (black arrow), blood vessel dilation (red arrow), and hemorrhage (yellow arrow) were found. It is noteworthy that the maximum blood vessel dilation was seen in the SB and Cit group. Abnormal cellular attachment (black arrow) in addition to hemorrhage (yellow arrow) was observed in the group that received SB and PS.

Histomorphometric analysis revealed a significant reduction in the thickness of the testicular tissue epithelium in the combined treatment groups (Cit + SB and SB + PS) when compared to the control group (*p* < 0.05). However, no significant changes were observed in the diameter of the seminiferous tubules across all treatment groups (Figure 6a,b). Furthermore, the counts of Leydig cells (Figure 7a), Sertoli cells (Figure 7b), and spermatogonia (Figure 7c) exhibited a marked decrease in all treatment groups relative to the control group (*p* < 0.05). Additionally, a reduction in the number of spermatocytes (Figure 7d) was noted in the Cit group, while the counts of spermatids (Figure 7e) significantly decreased in the Cit, PS, and Cit + SB groups compared to the control group (*p* < 0.05).

Figure 6Effects of food preservatives on (a) testis epithelial thickness and (b) seminiferous tubule diameter. Cit, citric acid; SB, sodium benzoate; PS, potassium sorbate.  ^∗^ *p* < 0.05 compared to the control group.

**Figure:**
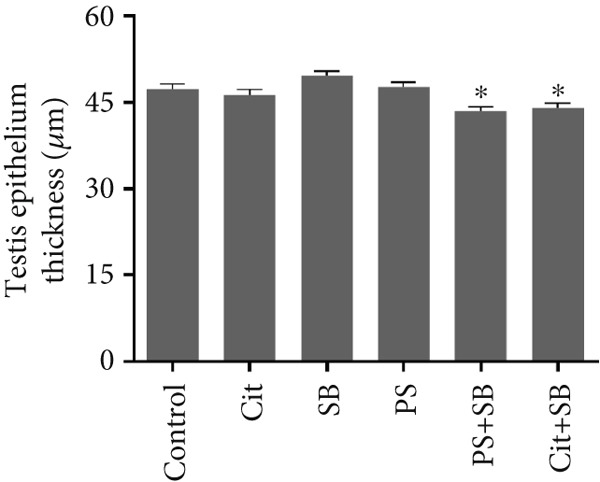
(a)

**Figure:**
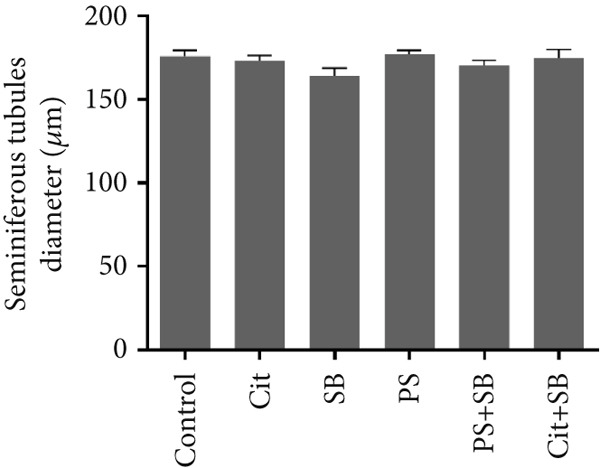
(b)

Figure 7Effects of food preservatives on number of (a) Leydig cells, (b) number of Sertoli cells, (c) number of spermatogonia, (d) number of spermatocytes, and (e) number of spermatids. Data are shown as mean ± SEM, *n* = 6. Cit, citric acid; SB, sodium benzoate; PS, potassium sorbate.  ^∗^ *p* < 0.05,  ^∗∗^ *p* < 0.01, and  ^∗∗∗^ *p* < 0.001 compared to the control group.

**Figure:**
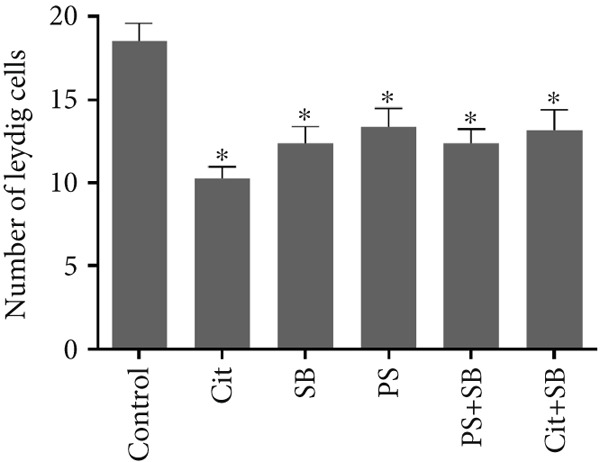
(a)

**Figure:**
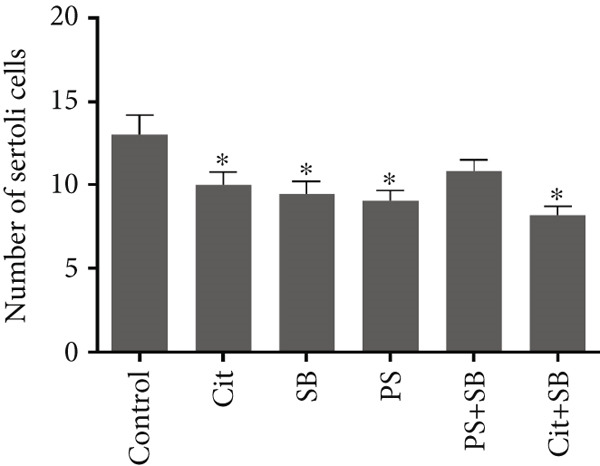
(b)

**Figure:**
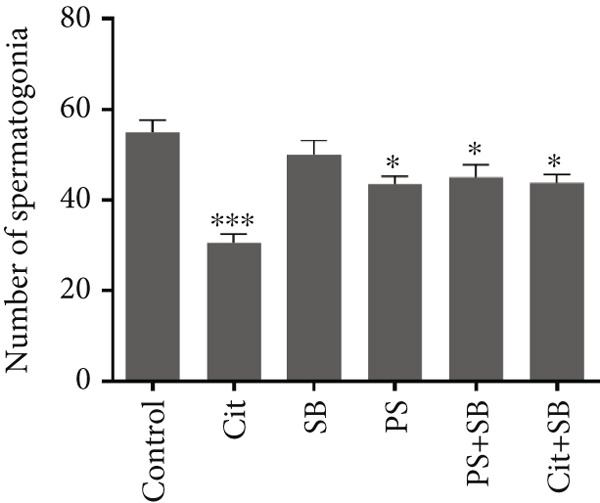
(c)

**Figure:**
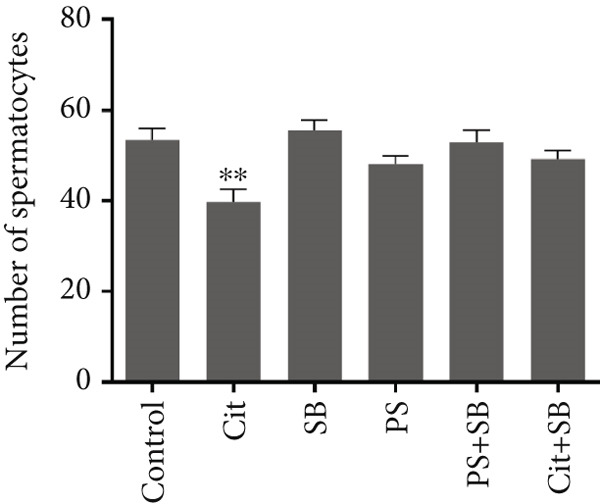
(d)

**Figure:**
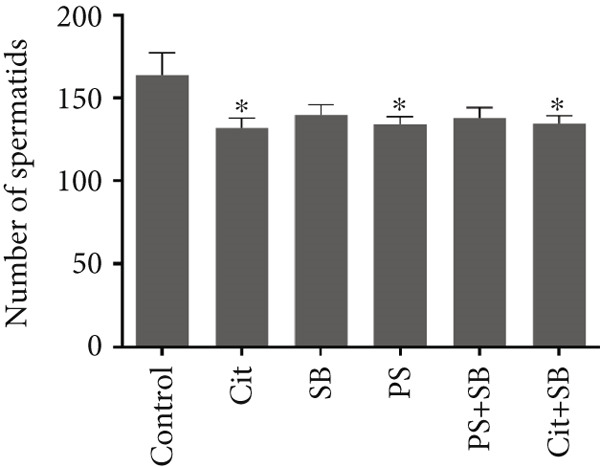
(e)

**Figure 8:**
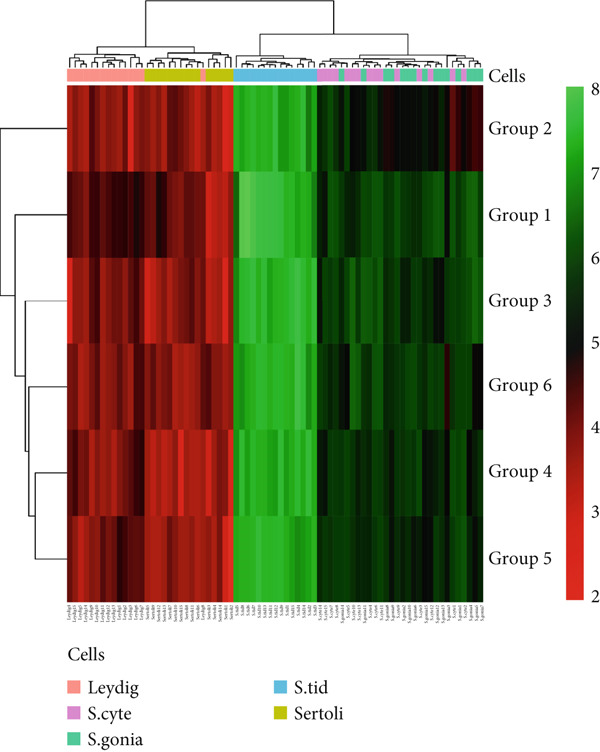
Hierarchical cluster analysis of the spermatogenic cells in the comparison between the experimental groups. Group 1, control; Group 2, citric acid; Group 3, sodium benzoate; Group 4, sodium benzoate + citric acid; Group 5, potassium sorbate; Group 6, sodium benzoate + potassium sorbate.

Hierarchical cluster analysis (Figure 8) of the spermatogenic cells revealed more similarity between groups control, SB, Cit + SB, PS, and SB + PS; meanwhile, group Cit was different. Additionally, more similarity was observed between the number of spermatogonia and spermatocytes, as well as the number of Sertoli and Leydig cells.

## 4. Discussion

This study demonstrated that SB and PS administration significantly reduced testicular and epididymal weight indices compared to controls. These findings align with previous research showing that reproductive toxicants commonly affect testicular weight through compromised spermatogenesis and oxidative stress [21].

The combination of SB and PS resulted in reduced sperm count, while PS alone and PS + SB negatively affected sperm viability and motility. These impairments may be linked to decreased testosterone levels and elevated MDA, suggesting compromised sperm membrane integrity, consistent with recent findings by Ogunro et al. [14].

Testosterone levels decreased significantly across all treatment groups, with Cit, PS, and PS + SB showing the most pronounced reductions. This decline is critical as testosterone maintains testicular structure and spermatogenesis [22].

The observed reduction in testosterone likely contributed to the decreased testicular weight and impaired sperm characteristics observed in this study.

While ROS levels remained stable, elevated MDA in PS, PS + SB, and Cit + SB groups indicated ongoing lipid peroxidation. This suggests that existing ROS levels were sufficient to trigger oxidative damage, possibly due to overwhelmed antioxidant systems [23].

The maintenance of total antioxidant capacity alongside elevated MDA levels indicates that while the system’s general antioxidant capacity was preserved, lipid‐specific protection was inadequate, leading to selective lipid peroxidation [24].

Apoptosis occurred in PS and PS + SB groups, confirmed by elevated Bax/Bcl‐2 ratios and caspase‐3 expression. The proapoptotic protein Bax promotes cytochrome C release, which activates caspase‐3 and initiates cell death [25]. This apoptosis correlated with decreased testosterone and testicular weight, suggesting that hormonal disruption contributed to testicular cell death.

Histological analysis revealed decreased testicular epithelial thickness with minor changes in seminiferous tubule diameter. This pattern typically indicates selective germ cell loss while preserving tubular architecture, often resulting from hormonal imbalances or oxidative stress affecting the germinal epithelium without major structural damage [26]. Decreased testosterone or FSH levels can impair maintenance of spermatogenesis, leading to loss of germ cells and thinning of the epithelium. However, Sertoli cells, which are less dependent on high levels of these hormones for survival, continue to support the basic architecture of the seminiferous tubules. As a result, the diameter of the tubules remains relatively unchanged, while the epithelium becomes thinner due to reduction in spermatogenic cells. Damage to Sertoli cells can disrupt spermatogenesis and thin the testicular epithelium yet the seminiferous tubules maintain their original shape. This occurs because Sertoli cells are less vulnerable to toxic damage than germ cells [27].

The reduction in spermatogonia, spermatocytes, and spermatids across different spermatogenic stages indicates comprehensive impairment of sperm production. Spermatogonia reduction suggests disrupted spermatogenesis initiation, while spermatocyte and spermatid decreases indicate problems in meiotic and differentiation phases, respectively [28].

These findings are consistent with El‐Shennawy et al. [2], who reported dose‐dependent reproductive toxicity of SB involving inflammation, oxidative stress, and apoptosis. Similarly, Adana et al. [13] demonstrated that PS alters semen parameters and testicular histoarchitecture in male rats, supporting our observations of reproductive impairment.

### 4.1. Limitations and Suggestions

This study was conducted over a specific timeframe and dose range. Long‐term exposure effects and dose–response relationships require further investigation. Additionally, molecular pathway analysis could provide deeper mechanistic insights. It is suggested that future studies examine the reversibility of these effects upon cessation of exposure and investigate protective interventions.

## 5. Conclusion

This study demonstrated the effects of three commonly used food preservatives, individually and in combination, on the male reproductive system in rats. The findings indicated that these chemicals can negatively impact sperm production, resulting in decreased viable and motile sperm, as well as significant changes in testicular histological appearance. Additionally, the preservatives may impair testicular function by causing lipid peroxidation, reducing testosterone levels, and inducing apoptosis. Notably, while Cit is classified as GRAS, the adverse effects identified in the male reproductive system in this study have not been previously documented for Cit acid treatment. Combined use of these preservatives may lead to reproductive issues in rats, raising concerns about their potential effects on human health. These results are consistent with previous studies on the adverse effects of preservative consumption; however, further research is needed to understand their impact on other critical systems and female reproductive health.

## Conflicts of Interest

The authors declare no conflicts of interest.

## Author Contributions

Marziyeh Haghshenas: writing—original draft, methodology, conceptualization, and formal analysis. Seyyed Sajad Daneshi: methodology, investigation, conceptualization, and formal analysis. Hassan Nategh Ahmadi: methodology and investigation. Samaneh Bina: methodology, investigation, and formal analysis. Fateme Esmaeilpoor: methodology and investigation. Razieh Bagheri: methodology and investigation. Mohammad Javad Khoshnoud: supervision and formal analysis. Azad Salimi: methodology and investigation. Seyedeh leili Asadi‐Yousefabad: methodology and formal analysis. Marzieh Rashedinia: design, writing—review and editing, supervision, project administration, funding acquisition, formal analysis, and conceptualization.

## Funding

This research was funded by Shiraz University of Medical Sciences (Grant No.: 31602, Ethics Code: IR.SUMS.REC.1402.051).

## Data Availability

The data that support the findings of this study are available from the corresponding author upon reasonable request.
